# Still's Disease in a Pediatric Patient after Liver Transplantation

**DOI:** 10.1155/2013/767684

**Published:** 2013-11-05

**Authors:** Juan-Carlos Meza, Evelyn Muñoz-Buitrón, Fabio Bonilla-Abadía, Carlos Alberto Cañas, Gabriel J. Tobón

**Affiliations:** ^1^Unit of Internal Medicine, Fundación Valle del Lili, CES University, Cali, Colombia; ^2^Clinical Investigative Unit, Fundación Valle del Lili, Cali, Colombia; ^3^Unit of Rheumatology, Fundación Valle del Lili, ICESI University, Cra 98 No. 18-49, Cali, Colombia

## Abstract

Still's disease (SD) is a multisystemic inflammatory disease characterized by persistent arthritis and in many cases with fever of unknown origin. Diagnosis of SD is challenging because of nonspecific characteristics and especially in the case of a patient with solid organ transplantation and immunosuppressive therapy where multiple causes of fever are possible. There is no diagnostic test for SD, even though some useful diagnostic criteria or laboratory findings, such as serum ferritin levels, have been proposed, and useful imaging studies for the diagnosis or followup of SD have not been developed. We report the case of a 9-year-old child who presented with high grade fever associated with joint pain after a history of liver transplantation and immunosuppressive therapy. Laboratory tests showed increased acute phase reactants, elevated ferritin, and leukocytosis. An 18 F-fluorodeoxyglucose positron emission tomography (18F-FDG PET) was performed identifying abnormal hypermetabolic areas localized in spleen, transplanted liver, and bone marrow secondary to inflammatory process. All infectious, autoimmune, and malignant causes were ruled out. A diagnosis of SD was performed and a steroid-based regimen was initiated with adequate response and no evidence of recurrence. To our knowledge this is the first case of SD following a solid organ transplant.

## 1. Introduction

Systemic juvenile idiopathic arthritis (JIA) or Still's disease (SD) is a multisystem inflammatory process that usually presents with high fever, a classic faint salmon-colored skin rash, arthritis, and variable systemic features like lymphadenopathy, serositis, odynophagia, and hepatosplenomegaly. The pathogenesis and etiology of SD have not yet been clearly determined [1]. Diagnosis of SD is challenging because of its low prevalence, heterogeneous clinical manifestations, and absence of pathognomonic clinical features [2]. Significant laboratory abnormalities include marked leukocytosis, thrombocytosis, and anemia in association with elevated acute phase reactants such as C-reactive protein (CRP), erythrocyte sedimentation rate (ESR), and serum ferritin which reflect an important systemic inflammatory response [3]. It is important to rule out a wide range of other diseases including infectious, malignant, and other rheumatic diseases, especially if fever occurs in patients with immunosuppressive conditions. Several studies have demonstrated the clinical value of using 18 F-fluorodeoxyglucose positron emission tomography (18F-FDG PET) scans to aid in the diagnosis of SD [4]. Here, we report a clinical case of SD in a pediatric patient after solid organ transplantation and immunosuppressive treatment. To our knowledge this is the first case showing these associations in both adults and pediatric patients.

## 2. Case Report

A 9-year-old child male from a rural area of Colombia was admitted with a clinical picture of one week of duration characterized by general symptoms, arthralgias, weakness, and high fever (40°C). Before admission to our institution, the patient was evaluated in a primary care center, where multiple tests were done. The initial tests showed leukocytosis with neutrophilia, high CRP levels, and a positive serum agglutination test for *Salmonella* Typhi. An antibiotic course with ceftriaxone was indicated. Then, we evaluated the patient in our center. The clinical chart was remarkable because a liver transplantation done five years ago related to liver failure due to hepatitis A. He was on immunosuppressive therapy with tacrolimus 1 mg BID and mycophenolate mofetil 250 mg 3 times per week. No steroid regimen was used at that time. On admission to our service, physical examination revealed a poor general condition, with fever, anterior cervical lymphadenopathy, and hepatosplenomegaly. Cardiopulmonary and neurological examinations were normal. A skin eruption was not evidenced.

**Figure 1:**
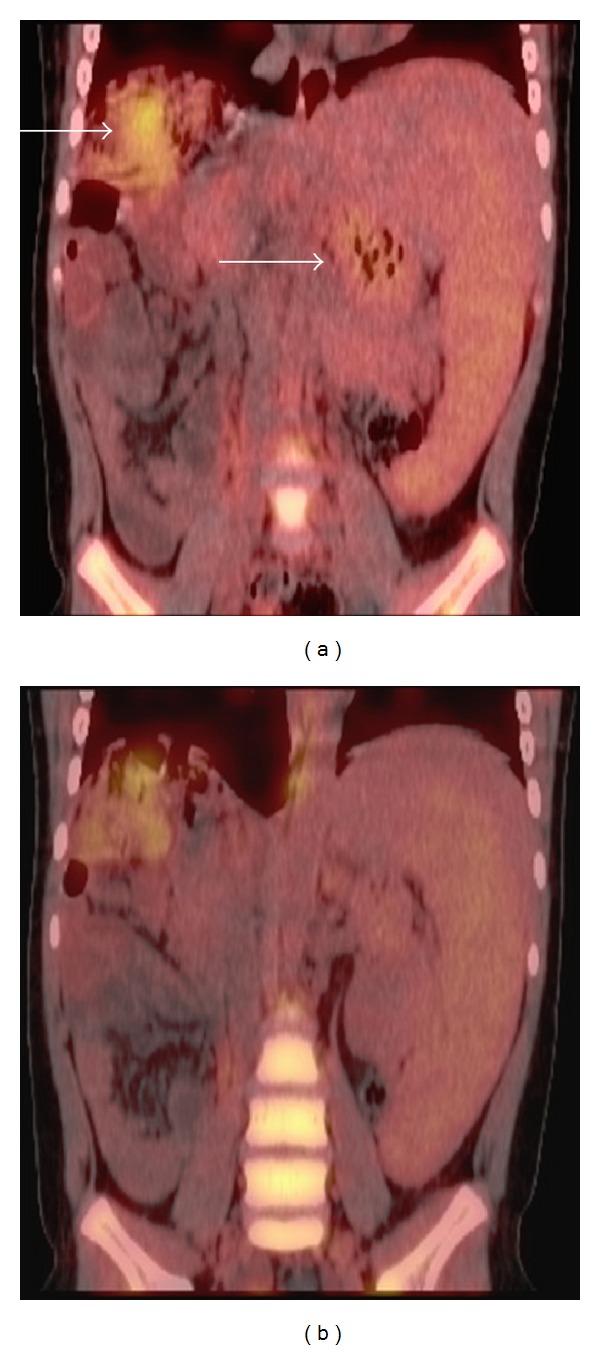
A PET scan identified abnormal hypermetabolic areas localized in spleen, transplanted liver, and bone marrow secondary to an inflammatory process (white arrows).

Extensive studies were completed to identify the cause of the fever. Laboratory test showed leukocytosis, neutrophilia, anemia (hemoglobin: 10.9 g/dL), normal platelets count and progressive increase of CRP up to 19.43 mg/dL, ESR of 76 mm/one hour, and serum ferritin levels of 2276 ng/mL (normal up to 300 ng/mL). Renal test, bilirubin level, aspartate aminotransferase, and alanine aminotransferase levels were all normal. Blood, urine cultures, and serology tests for *Cytomegalovirus* and *Epstein-Barr* were reported as negative. Autoimmunity tests revealed a rheumatoid factor of 15.3 UI/mL (cutoff 14 UI/mL), normal complement levels, and negative autoantibodies. Bone marrow examination to rule out infections or hematological malignancies, transthoracic echocardiogram, and paranasal sinuses computed tomography were all performed without abnormalities. Patient initially received empiric antibiotics (ceftriaxone and piperacillin-tazobactam) without clinical improvement. A PET scan (Figures 1(a) and 1(b)) identified abnormal hypermetabolic areas localized in spleen, transplanted liver, and bone marrow secondary to an inflammatory process. An ultrasound of liver and biliary tract documented splenomegaly. A diagnosis of systemic juvenile idiopathic arthritis (SD) was performed and a steroid-based regimen (1 mg per kg/day) was initiated with adequate response (clinical and biological evidence at 48 hours). Subsequent reductions in steroid dose were adequately tolerated, and biological findings showed improvement, with hemoglobin increasing levels (to 14 g/dL), and progressive decrease of CRP and ESR (0.09 mg/dL and 2 mm/h, resp.) was observed at 12 months followup without evidence of relapse.

## 3. Discussion

Systemic JIA is a disease clearly distinguished from all the other forms of JIA and very similar to adult-onset SD [2]. It is the most common childhood chronic rheumatic disease. Its incidence has been reported in high-income countries with around 2–20 cases per 100,000 population and a prevalence of 16–150 cases per 100,000 population [5]. SD may present with protean clinical manifestations like fever of unknown origin and many causes of this must be excluded before SD can be diagnosed definitively [1]. In the case of fever in solid organ transplant (SOT) patient another broad spectrum of etiologic causes must be considered. However, infections and malignancies as causes of fever of unknown origin have decreased whereas inflammatory diseases like collagen-vascular diseases, giant cell arteritis, rheumatoid arthritis, and other vasculitides have increased over time [6]. The diagnostics imaging has not proved useful because of the difficulty to differentiate SD from other pathologies. However, several studies have recently demonstrated the clinical value of using 18F-FDG PET scans to aid in the diagnosis of patients with unknown fever origin and SD and SOT patients [4, 7, 8]. We report an interesting case of SD where the utility of 18F-FDG PET is shown in the difficult diagnosis approach of a pediatric patient after SOT who presented primarily with fever of unknown origin. The relationship between SOT and SD development is difficult to explain and may not be explained in the light of this clinical case because the transplanted organ was not rejected and all of its functions were conserved. However, an interesting clinical observation derived from this case shows that the immunosuppressive treatment that our patient was receiving was not effective to avoid the development of SD.
